# SSRI use and clinical outcomes in epithelial ovarian cancer

**DOI:** 10.18632/oncotarget.8891

**Published:** 2016-04-21

**Authors:** Desiré K. Christensen, Guillermo N. Armaiz-Pena, Edgardo Ramirez, Koji Matsuo, Bridget Zimmerman, Behrouz Zand, Eileen Shinn, Michael J. Goodheart, David Bender, Premal H. Thaker, Amina Ahmed, Frank J. Penedo, Koen DeGeest, Luis Mendez, Frederick Domann, Anil K. Sood, Susan K. Lutgendorf

**Affiliations:** ^1^ Department of Psychological and Brain Sciences, University of Iowa, Iowa City, Iowa, USA; ^2^ Department of Pharmacology, Ponce Health Sciences University, Ponce, Puerto Rico; ^3^ Division of Gynecologic Oncology, Department of Obstetrics and Gynecology, University of Southern California, Los Angeles, California, USA; ^4^ Department of Biostatistics, University of Iowa, Iowa City, Iowa, USA; ^5^ Department of Gynecologic Oncology and Reproductive Medicine, UT MD Anderson Comprehensive Cancer Center, Houston, Texas, USA; ^6^ Department of Cancer Biology, and Center for RNA Interference and Noncoding RNA, UT MD Anderson Comprehensive Cancer Center, Houston, Texas, USA; ^7^ Department of Behavioral Science, UT MD Anderson Comprehensive Cancer Center, Houston, Texas, USA; ^8^ Department of Obstetrics and Gynecology, University of Iowa, Iowa City, Iowa, USA; ^9^ Holden Comprehensive Cancer Center, University of Iowa, Iowa City, Iowa, USA; ^10^ Division of Gynecologic Oncology, Department of Obstetrics and Gynecology, Washington University, St. Louis, Missouri, USA; ^11^ Department of Obstetrics and Gynecology, Lutheran General Hospital, Park Ridge, Illinois, USA; ^12^ Department of Medical Social Sciences, Northwestern University, Chicago, Illinois, USA; ^13^ Division of Gynecologic Oncology, Oregon Health and Sciences University, Portland, Oregon, USA; ^14^ Division of Gynecologic Oncology, Department of Obstetrics and Gynecology, Florida International University School of Medicine, Miami, Florida, USA; ^15^ Department of Radiation Oncology, University of Iowa, Iowa City, Iowa, USA; ^16^ Department of Urology, University of Iowa, Iowa City, Iowa, USA

**Keywords:** selective serotonin reuptake inhibitors, epithelial ovarian cancer, serotonin, progression, cell proliferation

## Abstract

Selective serotonin reuptake inhibitor (SSRI) use is common among ovarian cancer patients. We examined the effect of SSRIs on survival and progression in ovarian cancer patients and effects of 5-HT on ovarian cancer cell (OCC) proliferation. Ovarian cancer patients from a 6-site study between 1994 and 2010 were included. Cox proportional hazards models were used for multivariate analysis. SSRI use was associated with decreased time to disease recurrence (HR 1.3, CI 1.0-1.6, *p*=0.03), but not overall survival (HR 1.1, CI 0.9-1.3, *p*=0.56). Compared to normal ovarian cells, most OCCs had elevated 5-HT2A receptor mRNA expression (up to 1600 fold greater expression). Clonogenic survival increased in cells treated with 10 uM (1.6 fold, *p*<0.001) and 20uM (1.9 fold, *p*=0.018) 5-HT. Mice receiving 5-HT injections had increases in tumor weight (*p*=0.07) and nodules (*p*=0.08) with increased Ki67 expression. Injections with sertraline doubled mean tumor weight in mice (*p*=0.16). 5-HT and sertraline both increased Ki67 expression in mouse tumors (*p* < 0.001).

Patients using SSRIs had significantly decreased time to disease progression. It is possible that SSRIs alter serotonin levels in the tumor microenvironment, resulting in activation of proliferation pathways. Further characterization of serotonergic pathways in ovarian cancer is recommended to demonstrate safety of these medications.

## INTRODUCTION

Depression is common in ovarian cancer patients, with a recent study reporting that 55% of newly diagnosed patients suffer from clinical or subclinical depression. [1] Management of depression is important in oncology patients as it may negatively impact quality of life, [2, 3] adherence to medical regimens, [4] decision making [5] and cancer outcomes. [6, 7] Antidepressants are commonly prescribed to treat depression in cancer patients. Current clinically used antidepressants include but are not limited to selective serotonin-reuptake inhibitors (SSRIs), serotonin norepinephrine-reuptake inhibitors (SNRIs), and tricyclic antidepressants (TCAs). [8] It is unclear how best to manage depression in cancer populations since there have been reports that antidepressants, specifically SSRIs and TCAs, may promote tumor growth in preclinical experimental models for various cancers. [9–12]

Animal experiments have demonstrated inhibition of lymphocyte proliferation and growth-promoting effects of SSRIs on mammary tumors, melanoma, and fibrosarcoma. [13] However, results from experimental studies are inconsistent. [14] Fluoxetine (20-80 mg/kg) promoted fibrosarcomas (mice), melanomas (mice) and mammary carcinogenesis (rats). [13, 14] In contrast to these findings, fluoxetine (10-20 mg/kg) demonstrated antineoplastic effects on chemically induced colonic tumors in rats. [14] Fluoxetine (30mg/kg) has also been shown to reduce colon cancer development in rats. [12] The potential for antidepressants to promote tumor growth has led to various epidemiologic studies examining the risk of developing cancer among antidepressant users. There have been inconsistent findings with respect to risk of developing ovarian cancer among antidepressant users, [15–22] although meta-analysis revealed a modest increased risk of breast and ovarian cancer with the use of antidepressants, especially SSRIs. [22]

Antidepressants have been designed to alter serotonergic pathways. Serotonin (5-hydroxytryptamine (5-HT)) performs a variety of functions as a neurotransmitter with roles in mood, homeostatic regulation, and intestinal motility. Many of its functions in the periphery are relevant to tumor development. 5-HT has shown mitogenic effects on several carcinomas including breast cancer cells [23], human pancreatic carcinoid cells [24], rat colonic adenocarcinoma [25] and human small cell lung carcinoma [26]. 5-HT signaling is initiated through interaction with a receptor subtype from one of seven receptor families, the majority of which are G-coupled transmembrane proteins from families 1 and 2. [27] Immunohistochemistry has revealed the presence of 5-HT receptors in several tumor types, including ovarian. [28]

Peripheral 5-HT in the blood is carried by platelets and released in high concentrations (micromolar) during degranulation events. [29] Platelets possess the serotonin transporter (SERT), allowing them to uptake 5-HT across their plasma membrane with further transport into dense granules via a monoamine transporter. [30] Selective-serotonin reuptake inhibitors (SSRIs) are designed to increase 5-HT levels in the synaptic cleft by inhibiting SERT reuptake of 5-HT. Outside of their designed function, SSRIs decrease reuptake of 5-HT by platelets through binding of SERT. [30, 31] Consequences of changes to peripheral 5-HT regulation are poorly understood and have important implications regarding tumor growth in cancer patients.

To date, no studies have examined effects of SSRIs among patients already diagnosed with ovarian cancer and the influence SSRIs may have on survival and disease progression in these patients. Moreover, no studies have explored the effect of 5-HT or SSRIs on ovarian neoplastic cell growth. To address these knowledge gaps, we characterized 5-HT receptor expression in ovarian cancer cells and investigated the functional and biological roles of 5-HT using *in vitro* and *in vivo* models. In addition, this multicenter study examined effects of SSRIs on survival and disease progression in patients diagnosed with epithelial ovarian cancer.

## RESULTS

### Patient characteristics

Demographic and clinical characteristics of the sample are listed in Table 1. The median patient age was 60.1 (IQR, 52 to 68) years. The median follow-up duration was 56.4 (IQR, 31.2 to 85.2) months for all patients: 60.0 (IQR, 34.8 to 86.4) months for antidepressant users and 62.4 (IQR, 39.6 to 82.8) months for SSRI users. Patients predominantly had advanced stage (82%) and high-grade (81%) disease, with serous histology (76%). A majority of patients (64%) had received optimal cytoreduction. Antidepressants were used by 26% of patients. The most commonly used class of antidepressants was SSRIs (16%) followed by SNRIs (6%), TCAs (3%). Sertraline was the most commonly prescribed SSRI (n=34), followed by fluoxetine (n=30), escitalopram (n=25), paroxetine (n=23) and citalopram (n=18). Four percent of patients (n=28) used two different antidepressant classes. Only SSRIs were analyzed separately due to small sample size for other classes of antidepressants. Comparison of patient characteristics showed that antidepressant users were more likely to be white (97% vs. 87%, *p*<0.001) and had a slightly lower percentage with suboptimal residual disease (31% vs. 38%, *p*=0.07) than patients not using antidepressants. There was no significant difference in age (*p*=0.16), stage (*p*=0.84), grade (*p*=0.11), histology (*p*=0.74), or duration of follow-up (*p*=0.19). Similar results were observed comparing characteristics of SSRI users and patients not using SSRIs.

**Table 1:** Patent characteristics

	All patients (N=773)		Patients on ADs (n=203)		Patients on SSRIs (n=125)	
Characteristic	No.	%	No.	%	No.	%
Age (years)						
Median	60.1		59.0		59.7	
IQR	52-68		50.3-67		51-67	
FU duration, months						
Median	54.8		58.8		62.5	
IQR	31.4-85.3		33.5-83.2		37.7-83.2	
Race						
White	679	91.6	183	97.3	115	97.5
Other	62	8.4	5	2.7	3	2.5
Stage						
I	78	10.2	23	11.3	16	12.8
II	60	7.8	15	7.4	10	8.0
III	549	71.9	147	72.4	88	70.4
IV	77	10.1	18	8.9	11	8.8
Residual Disease						
Optimal	492	63.7	140	69.0	87	69.6
Suboptimal	281	36.4	63	31.0	38	30.4
Grade						
High	627	81.4	173	85.2	105	84.0
Low	143	18.6	30	14.8	20	16.0
Histology						
Serous	582	75.5	155	76.4	93	74.4
Other	189	24.5	48	23.6	32	25.6
ADs	203	26.3	203	100.0	125	100.0
SSRIs	125	16.2	125	61.6	125	100.0
SNRIs	44	5.7	44	21.7	7	5.6
TCAs	24	3.1	24	11.8	3	2.4
NDRIs	18	2.3	18	8.9	5	4.0
SARIs	11	1.4	11	5.4	3	2.4
NaSSAs	9	1.2	9	4.4	3	2.4

Abbreviations: ADs, antidepressants; FU, follow-up; IQR, interquartile range; NaSSAs, noradrenergic and specific serotonergic antidepressants; NDRIs, norepinephrine-dopamine reuptake inhibitors; SARIs, serotonin antagonist-reuptake inhibitors; SNRIs, selective norepinephrine-reuptake inhibitors; SSRIs, selective serotonin-reuptake inhibitors; TCAs, tricyclic antidepressants.

### Univariate survival analyses

Univariate analyses indicated that age, stages III and IV disease, suboptimal cytoreduction, high grade and serous histology were significantly associated with both shorter survival time and time to disease recurrence (Supplementary Table S2). Neither use of antidepressants nor use of SSRIs was significantly associated with survival or progression.

### Multivariate model

Cox multivariate regression was performed to examine adjusted risks of survival in ovarian cancer patients. Adjusting for disease stage, grade, histology, residual disease, and age, there was no difference in time to death or progression between patients using vs. not using antidepressants (all *p*-values > .50) (Table 2). Similarly, there was no difference in overall survival between patients using vs. not using SSRIs (*p*=0.56). However, multivariate analysis did reveal a significantly decreased time to disease progression in SSRI users vs. non-users (HR=1.3, 95% CI 1.0-1.6; *p*=0.03). In addition, logistic regression revealed a possible trend towards an increased risk of progression in SSRI users (OR 1.44, CI 0.9-2.3, *p*=0.12), although not statistically significant.

**Table 2:** Multivariate cox proportional hazard ratios for antidepressant use, overall survival, and PFS in ovarian cancer patients

Covariate	All ADs						SSRIs					
	Progression			Death			Progression			Death		
	HR	95% CI	*P*	HR	95% CI	*P*	HR	95% CI	*P*	HR	95% CI	*P*
Age												
(unit = 10 yrs)	1.1	1.1-1.2	0.20	1.2	1.1-1.3	<0.001	1.1	1.0-1.1	0.20	1.2	1.1-1.4	<0.001
Stage *v* I												
II	1.6	0.9-2.9	0.09	1.1	0.5-2.4	0.83	1.6	0.9-2.9	0.09	1.1	0.5-2.5	0.80
III	3.9	2.5-6.1	<0.001	3.2	1.8-5.9	<0.001	4.0	2.5-6.2	<0.001	3.3	1.8-5.9	<0.001
IV	5.5	3.3-9.2	<0.001	4.9	2.5-9.3	<0.001	5.6	3.3-9.3	<0.001	5.0	2.6-9.5	<0.001
RD *v* optimal												
Suboptimal	1.9	1.6-2.3	<0.001	2.1	1.7-2.6	<0.001	2.0	1.6-2.4	<0.001	2.1	1.7-2.6	<0.001
Grade *v* low												
High	1.2	0.9-1.5	0.16	1.1	0.9-1.5	0.34	1.2	0.9-1.5	0.19	1.1	0.9-1.5	0.38
Histol *v* other												
Serous	1.1	0.9-1.4	0.27	1.0	0.8-1.4	0.73	1.1	0.9-1.4	0.26	1.0	0.8-1.4	0.77
AD *v* non-use												
Use	1.1	0.9-1.3	0.51	1.0	0.8-1.2	0.70	1.3	1.0-1.6	0.03	1.1	0.8-1.4	0.56

Abbreviations: AD, antidepressant; Histol, histology; PFS, progression-free survival; RD, residual disease; SSRI, selective serotonin-reuptake inhibitor; yrs, years.

### 5-HT receptor expression and proliferation

**Figure 1:**
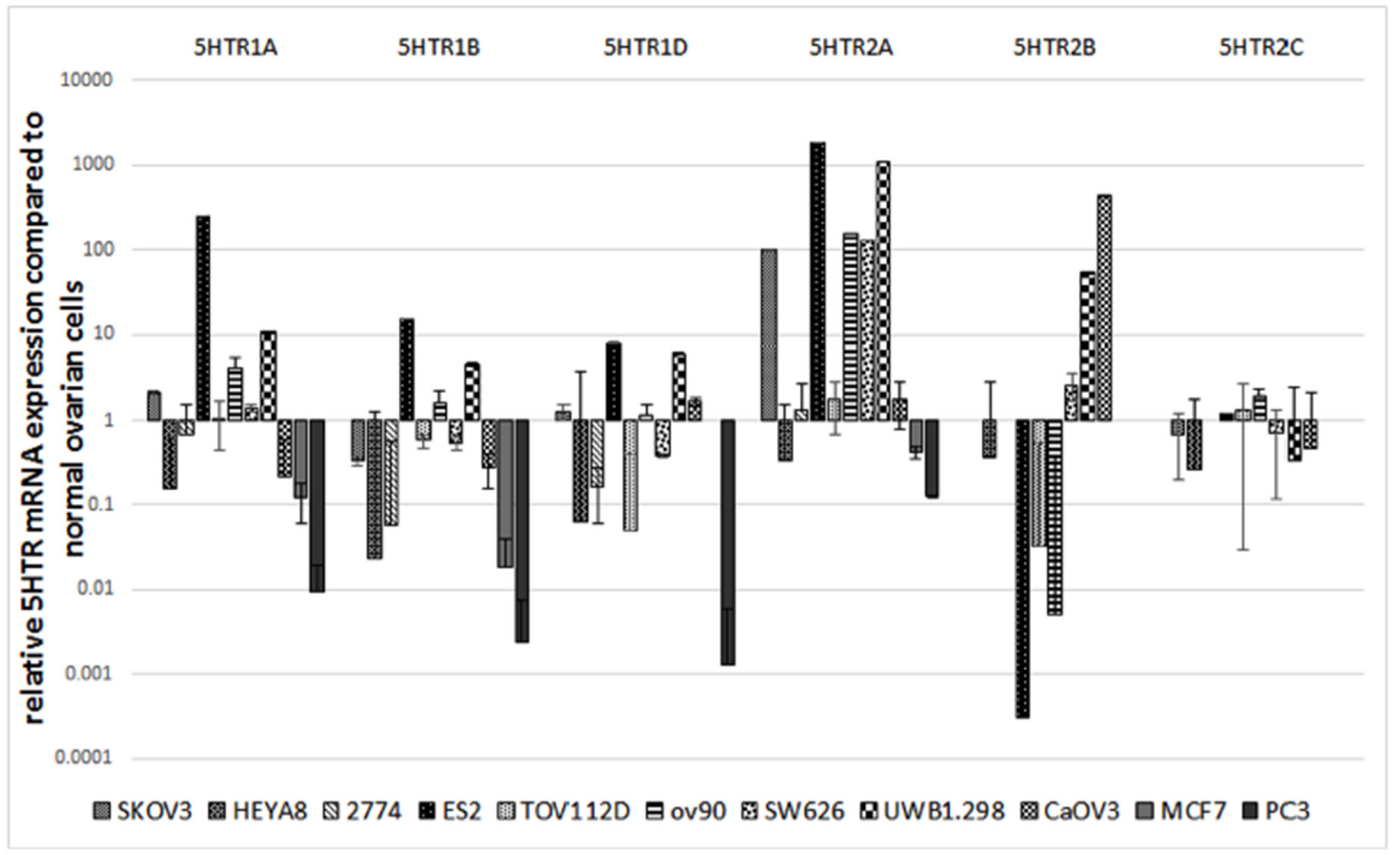
Relative 5HTR (families 1 and 2) mRNA expression of 9 OCC lines (SKOV3, HEYA8, 2774, ES2, TOV112D, OV90, SW626, UWB1.298 and CaOV3) compared to normal ovarian cells on a log scale Compared to normal ovarian cells, 8 out of 9 OCC lines had elevated 5HTR2A mRNA expression (up to 1600 fold greater expression). Results represented as the mean +/− SEM.

Given the association of SSRI use with shorter time to disease progression, we next asked whether cancer cells could be impacted directly by SSRIs. All OCC lines expressed mRNA for 5-HT receptor subtypes 1A, 1B, 1D, and 2A (Figure 1). Only 2 OCCs (2774 and CaOV3) expressed subtype 1E mRNA; 1F was not observed in any of the cell lines investigated. Compared to normal ovarian cells, 8 out of 9 OCCs had elevated 5-HT2A receptor mRNA expression (up to 1600 fold greater expression; p-values <0.001).

**Figure 2:**
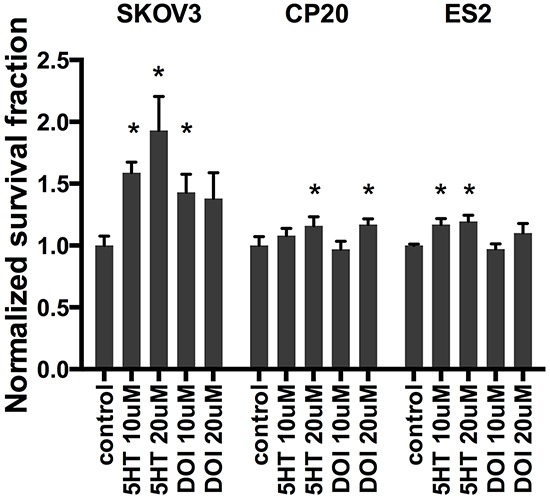
Clonogenic survival assays in SKOV3, CP20 and ES2 cells Increased SKOV3 proliferation was observed after treatment with 10 uM (1.6 fold, *p*<0.001) and 20 uM (1.9 fold, *p*=0.018) 5-HT. DOI increased SKOV3 survival at 10 uM (1.4 fold, *p*=0.014) and 20 uM (1.4 fold, *p*=0.14) doses. CP20 cell proliferation increased with 20 uM 5-HT (1.16 fold, *p*<0.001) and 20 uM DOI (1.17 fold, *p*<0.001) but was not changed by 10 uM 5-HT (*p*=0.79) or 10uM DOI (*p*=0.18). ES2 cell proliferation was increased by 10 uM 5-HT (1.17 fold, *p*<0.01) and 20 uM 5-HT (1.20 fold, *p*<0.01). ES2 cell proliferation was not changed by 10 uM DOI (*p*=0.43) and 20 uM (*p*=0.13). Results represented as the mean +/− SEM.

Clonogenic survival was significantly increased in SKOV3 cells (Figure 2) after treatment with 10 uM (1.6 fold, *p*<0.001) and 20 uM (1.9 fold, *p*=0.018) 5-HT. DOI, a potent 5HT2A receptor agonist, increased clonogenic survival in SKOV3 cells at 10 uM (1.4 fold, *p*=0.014) and 20 uM (1.4 fold, *p*=0.14) doses. Survival was also significantly increased in CP20 cell proliferation after treatment with 20 uM 5-HT (1.16 fold, *p*<0.001) and 20 uM DOI (1.17 fold, *p*<0.001) but was not changed by 10 uM 5-HT (*p*=0.79) or 10uM DOI (*p*=0.18). ES2 cell proliferation was increased by 10 uM 5-HT (1.17 fold, *p*<0.01) and 20 uM 5-HT (1.20 fold, *p*<0.01). ES2 cell proliferation was not changed by 10 uM DOI (*p*=0.43) and 20 uM (*p*=0.13). The magnitude of increased survival was greatest in SKOV3 cells compared to CP20 and ES2 cells. ES2 cell survival demonstrated an increasing trend of survival with 20uM DOI, but the results were not significant.

### *In vivo* mouse experiments

**Figure 3:**
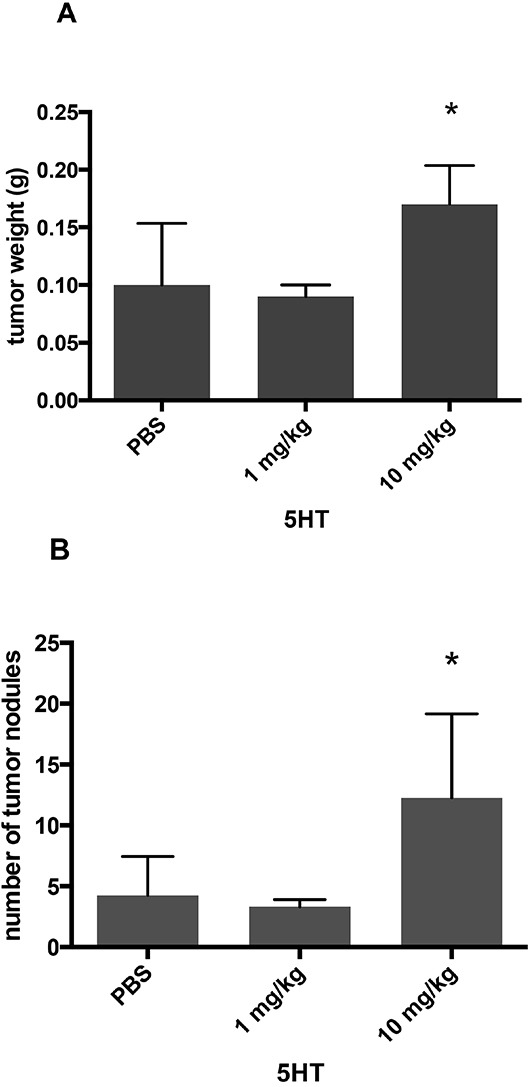

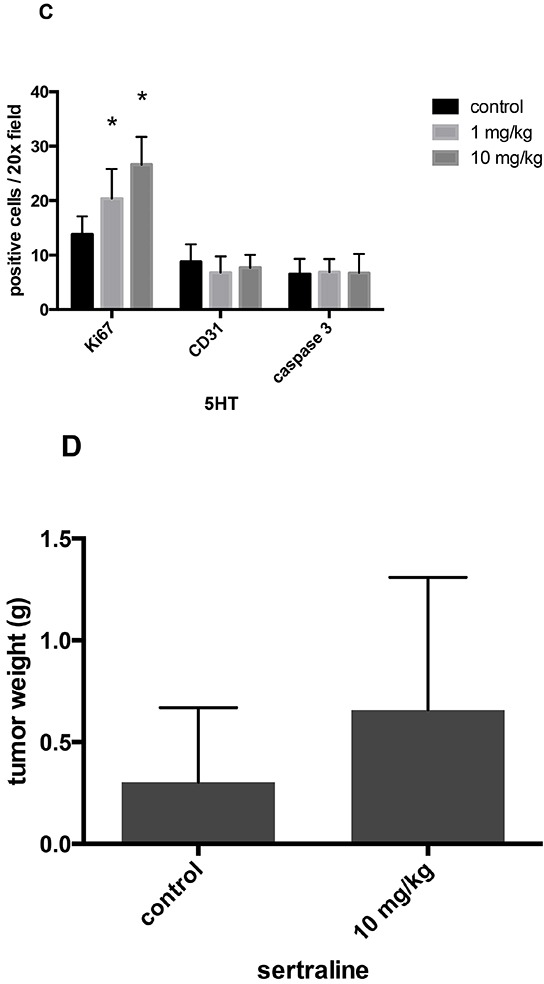

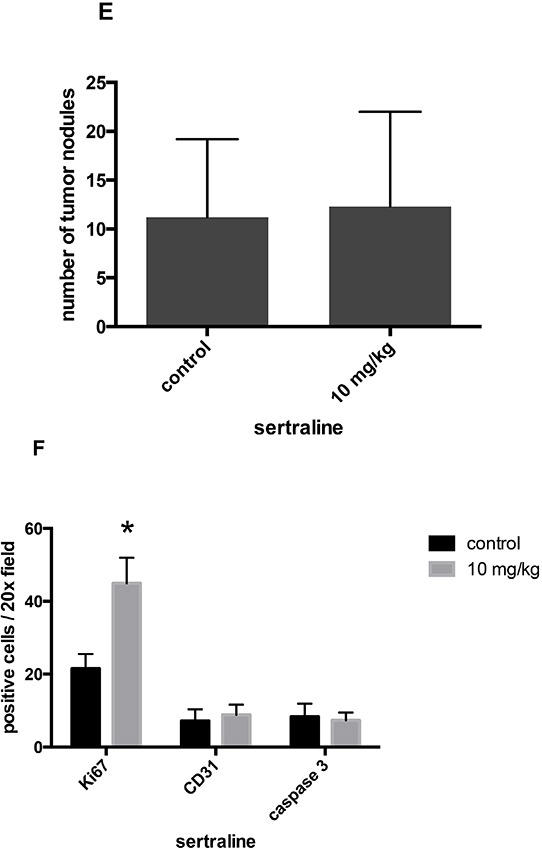
*In vivo* mouse experiment in 8- to 12- week old female athymic nude mice administered daily 5-HT or sertraline injections **A.** 5-HT 10mg/kg injections increased tumor weight (*p*=0.07). **B.** 5-HT 10 mg/kg injections increased number of nodules (*p*=0.08). **C.** Ki67 was significantly increased at 1 mg/kg (*p*<0.001) and 10 mg/kg (*p*<0.001) 5-HT. **D.** Sertraline 10 mg/kg injections increased tumor weight 2-fold (*p*=0.16). **E.** Sertraline injections at 10 mg/kg was not associated with an increase in number of tumor nodules. **F.** Ki67 was significantly increased with 10 mg/kg (*p*<0.001) sertraline injection. Results represented as the mean +/− SE.

To examine potential *in vivo* effects of 5-HT and sertraline, we used an orthotopic mouse model of ovarian cancer. Sertraline was selected for *in vivo* experiments because it was the most commonly used antidepressant in our patient sample. Following treatment with either 5HT or sertraline, mice received an intraperitoneal injection of SKOV3 cancer cells. 5-HT 10 mg/kg injections increased tumor weight (*p*=0.07) (Figure 3A) and number of nodules (*p*=0.08) (Figure 3B). No difference was observed with 1 mg/kg doses. Consistent with our *in vitro* findings, Ki67 was significantly increased by both 1 mg/kg (*p*<0.001) and 10 mg/kg (*p*<0.001) dosing with no change observed in CD31 or caspase 3 (Figure 3C). Tumor weight doubled after 10 mg/kg sertraline injections, although not statistically significant (*p*=0.16) (Figure 3D) with no change seen in number of tumor nodules (*p*=0.78) (Figure 3E). Ki67 was significantly increased by 10 mg/kg (*p*<0.001) dosing with no change observed in CD31 or caspase 3 (Figure 3F).

## DISCUSSION

Key findings of this study are that SSRIs are associated with a decreased time to disease progression after adjusting for significant covariates, although they are not associated with overall survival. The effect size of 5-HT on cell survival was variable depending on the cell line studied. There is substantial variability in patient tumor composition between individuals that could account for some of the insignificant and modestly significant findings described in this study. These findings are the first to indicate the use of SSRIs among ovarian cancer patients may impact disease progression. The present study is unique as the majority of studies on antidepressant use have focused on its relationship to risk of developing cancer. A recent meta-analysis of 61 studies examined the relationship between antidepressants and the risk of developing breast or ovarian cancer and reported a small but statistically significant increase in the risk for breast and ovarian cancer in women who use SSRIs. [22] The effects of antidepressants on survival and progression have been minimally examined in clinical populations; existing studies have tended to focus on breast cancer patients concurrently using tamoxifen. [37] Tamoxifen has been explored as an ovarian cancer therapy, [38, 39] and inhibition of CYP2D6 by some SSRIs (most notably fluoxetine and paroxetine) could reduce effective tamoxifen utilization in ovarian cancer patients through decreased generation of active metabolites. Through drug interactions and direct effects on the tumor cells, there may be specific implications to antidepressant selection in cancer patients.

5-HT was shown to have a mitogenic effect on OCCs that was partially mediated by the 5-HT2A receptor. 5-HT2A receptor mRNA was upregulated in OCCs compared to normal ovarian epithelial cells, supporting a physiologic role of 5-HT in OCC growth. *In vivo* results demonstrated increased tumor burden with upregulation of Ki67, indicating 5-HT signaling is coupled to proliferation. We also demonstrated that sertraline promotes cell proliferation by upregulating Ki67. In human endothelial cells, 5-HT has been shown to activate angiogenic phosphorylation signaling. [40] Neither 5-HT or sertraline increased CD31, an angiogenic marker, or caspase 3, apoptotic marker, in our study. Both 5-HT and sertraline demonstrate mitogenic effects on ovarian neoplastic cells through similar cellular signaling pathways. Our results support a mechanism by which SSRIs may promote growth of residual neoplastic disease through altered 5-HT levels in the tumor microenvironment, thereby decreasing time to disease progression.

Growing evidence supports the involvement of 5-HT and SSRIs in immune system interactions. 5-HT is also an important mediator of immune cell signaling, promoting neutrophil recruitment [41] and cell signaling of mast cells and lymphocytes. [42, 43] SSRIs have been shown to depress immune cell function, largely suspected to be the result of decreased platelet 5-HT concentrations. After 3 weeks of effective dosing, SSRIs decrease blood serotonin content by 90% and inhibit platelet reuptake of 5-HT. [44] There is accumulating evidence that platelet release of 5-HT is involved in inflammatory processes, including joint edema. [45] Suppressed lymphocyte proliferation was observed after SSRI administration *in vivo*. [46] By compromising optimal immune function, SSRIs may allow tumor cells to evade detection and cytotoxic killing.

This study contributes to the growing body of research supporting further characterization of serotonergic effects of medications in cancer patients. These findings are potentially relevant for other classes of medications. Numerous other serotonergic agents are commonly used in ovarian cancer patients, such as certain analgesics (e.g. tramadol), antibiotics (e.g. linezolid) and antihistamines (e.g. diphenhydramine). These medications have the potential to affect 5HT levels and SERT activity. The clinical relevance of their serotonergic effects on tumor biology is currently unknown. Much like monoamine oxidase inhibitors (MAOIs), linezolid inhibits monamine oxidase nonselectively increasing neurotransmitter levels. Other neurotransmitters have been shown to have systemic functions relevant to tumor biology. Tumor growth has been shown to be promoted by norepinephrine and inhibited by dopamine. [47, 48] Selective norepinephrine-reuptake inhibitors (SNRIs), tricyclic antidepressants (TCAs), norepinephrine-dopamine reuptake inhibitors (NDRIs) and noradrenergic and specific serotonergic antidepressants (NaSSAs) increase levels of norepinephrine, and NDRIs increase levels of dopamine. It is unknown how antidepressants affect the levels of these neurotransmitters at the tumor site. The systemic impact of antidepressants and their influence on the tumor microenvironment are important considerations for future research.

### Strengths

This is the first study to examine the affects of antidepressants on progression and survival in ovarian cancer patients. Data were compiled from multiple medical centers resulting in a large sample size with adequate power to detect clinically significant differences. Antidepressant use at time of surgery was available through medical records reducing the possibility of recall bias.

### Limitations

An important limitation of this study is the lack of information on duration and dose of antidepressant use. Patients were classified as antidepressant users or non-users based on medical record information at the time of surgery. Information on previous use of antidepressants and length of use was not available. This study also did not explore dose effects of antidepressants on ovarian cancer patient outcomes, and this is an important question for future research. The study was unable to account for discontinued use or *de novo* use of antidepressants during the follow-up period. Information on the success of antidepressant treatment was also unavailable. Unsuccessful treatment of depression could contribute to poor outcomes in cancer patients. This study is unable to differentiate between risk of death from antidepressant use versus depression.

This study found that ovarian cancer patients using SSRIs had decreased time to disease progression. At this time, more data on duration of antidepressant use in diagnosed cancer populations would be important to further clarify these findings. More research is recommended to understand the mechanisms behind the possible contribution of SSRIs to circulating serotonin levels and influence on the tumor microenvironment. Examination of associations between cancer survival and different classes of antidepressants, including SNRIs, TCAs, and aminoketones (such as bupropion) also have potential clinical relevance. There may be direct drug effects as well as modifications to serotonin levels that contribute to risk of cancer recurrence.

## MATERIALS AND METHODS

### Patient data

#### Study population

Records of women (n=773) diagnosed with ovarian cancer at six study sites (University of Iowa Hospital and Clinics (n=192), University of Miami (n=21), Mercy Medical Center (Miami) (n=2), Washington University (n=12), MD Anderson Cancer Center (n=270), and Mercy Medical Center Baltimore, MD (n=276)) were examined. Approval from institutional review boards of all recruitment sites was obtained prior to patient enrollment, and all participants provided written informed consent. Ovarian cancer was confirmed by histological diagnosis of primary invasive epithelial ovarian, peritoneal or fallopian tube cancer (stages I through IV were included) at time of upfront surgical debulking. A minimum of 6-month patient follow-up was required for inclusion in the study. Patients were excluded if another organ site was the location of the primary cancer or if the primary cancer was not epithelial. At the Iowa, Miami, and Washington University sites, where accrual was part of a larger investigation of psychosocial factors and biomarkers, patients were excluded if they had used systemic corticosteroid medications in the month before surgery, if they had comorbidities known to alter the immune response, or were under 18 years of age.

Clinical information including age, grade, stage, histology, extent of cytoreduction, antidepressant use, progression and survival was obtained from medical records. Antidepressant use was based on current medication use at the time of surgery. Patient recruitment and censoring occurred over the different dates based on site: Universities of Iowa and Miami and Mercy Medical Center (Miami) from 2003 to 2010 (censored June 2011), Washington University from 2009 to 2010 (censored June 2011), MD Anderson from 1994 to 2008 (censored December 2010), and Baltimore from 1994 to 2009 (censored May 2011). The longest follow-up duration was 18 years for the earliest recruited Baltimore patients. Progression-free survival was defined as the time between surgery and the first recurrence (if complete response), or progression, as defined by GCIG and RECIST criteria. [32] Overall survival was measured from the date of surgery until date of death. [33] Information was missing on death for 3 patients and on progression for 7 patients.

#### Statistical analysis

SAS 9.3 was used for all analyses of the clinical study data. Descriptive patient characteristics were compiled and differences examined using *X* ^2^ and Fisher's exact tests for categorical variables and independent samples *t* tests for continuous variables. Median follow-up duration was calculated using the inverse Kaplan-Meier method. Two sets of analyses were performed on progression and survival; one tested use of all antidepressants with progression and survival, the second tested use of SSRIs with progression and survival. The association of survival time with antidepressant use and with each potential covariate was examined using univariate Cox proportional hazard regression models. [34] From the fitted Cox models, the unadjusted hazard ratio with 95% confidence intervals was obtained, and significance of the association was tested using the Wald Chi-square statistic. Covariates included in univariate and multivariate analyses were stage (advanced-stage: III, IV vs. early-stage: I, II), grade (high vs. low), residual disease (suboptimal vs. optimal cytoreduction <1cm), histology (serous vs. non-serous), and age. Standard diagnostics were utilized to evaluate model adequacy. [35] Statistical significance was set at *p* < 0.05 and all analyses were two-tailed.

#### Power analysis

With the study sample size, there is an 80% power to detect an 8.5% decline in time to disease progression with use of antidepressants and 10% decline in time to progression with use of SSRIs. There is an 80% power to detect a 9.5% decline in survival with use of antidepressants and 11% decline in survival with use of SSRIs.

#### Cell culture

Ovarian carcinoma cell lines (A2780-CP20, SKOV3, HEYA8, 2774, ES2, TOV112D, OV90, SW626, UWB1.298 and CaOV3) and normal ovarian cells were cultured in RPMI-1640 media supplemented with 15% fetal bovine serum and 0.1% gentamicin. A2780-CP20 (labeled CP20) cells were developed by sequential exposure of A2780 cells to cisplatin of increasing concentrations. Cells were obtained from the institutional Cell Line Core laboratory and authentication performed once per year according to institutional policy (MD Anderson ACA#1044) using the Promega Power Plex 16HS kit (Promega). Mycoplasma screening was performed with the MycoAlert Kit (Lonza). Cells were maintained in an incubator at 37°C in 5% CO_2_.

#### Real-time RT-PCR assays for gene expression

Quantitative real-time RT-PCR was used to determine 5HT receptor mRNA expression. Total cellular RNA was isolated using RNeasy Mini Kit (Qiagen, Valencia, CA, USA) and quantified using a biophotometer (Eppendorf, Westbury, NY, USA). Reverse transcription to yield cDNA for real-time RT-PCR analysis was performed using a High Capacity cDNA Archive Kit (Applied Biosystems Inc., Foster City, CA, USA). The reverse transcription reaction of 2 ug of RNA was primed with random primers and incubated at 25C for 10 min followed by 37C for 120 min. The primer/probe PCR reactions consisted of 80 ng of cDNA added to 12.5 ul of TaqMan Universal PCR Master Mix (Applied Biosystems Inc.), 1.25 ul gene-specific primer/probe mix, and 6.25 uL PCR grade water, for a 25 uL total reaction. Primers for all of the serotonin receptor subtypes in families 1 and 2 were designed using Roche Universal ProbeLibrary and ABI primer express software (Supplementary Table S1). The PCR conditions for all reactions were 95C for 10 min, followed by 40 cycles of 95C for 15s, with annealing at 60C for 1 min. Real-Time PCR was performed on an ABI 7000 real-time sequence detection system. Gene-specific TaqMan probes were labeled with a 5′ reporter dye, 6-FAM, and a 3′ end containing a nonfluorescent quencher and a minor groove binder. Fold differences in OCC mRNA expression relative to normal ovarian cells were calculated using their respective mRNA expression calibrated to 18-s ribosomal RNA expression and computed using ABI relative quantitative software (Applied Biosystems Inc.). MCF-7 and PC3 cells served as positive controls for 5HT2A [23] and 5HT1A and B [36] receptor expression respectively.

#### Clonogenic survival assays

On 60mm tissue culture dishes, cells (SKOV3, CP20 and ES2) were plated to reach 80 percent confluency after 72 hours. After 48 hours, cells were treated with 5-HT or 2,5-dimethoxy-4-iodoamphetamine (DOI), a potent 5HT2A receptor agonist, using one of the following conditions: culture media (untreated), 10 uM 5-HT, 20 uM 5-HT, 10 uM DOI, or 20uM DOI. Cells were incubated in culture media, 5HT or DOI for 24 hours. Cells were then treated with TrypLE Express (1X), counted with a hemacytometer and then plated at concentrations of 500 and 1000 cells per plate. Colonies were grown for 14 days and then stained with Coomassie. Colonies >50 cells were counted to determine the normalized surviving fraction (NSF). Surviving fraction (SF) = (colonies counted)/(cells plated); NSF = (SF of the clonogenic survival plate)/(average SF of fraction of untreated plates). Each condition was performed in triplicate and replicated 3 times.

#### *In vivo* mouse experiments

Female athymic nude mice between 8 to 12 weeks old were obtained from the National Cancer Institute. All experiments were approved by the Institutional Animal Care and Use Committee at M. D. Anderson Cancer Center. Mice were administered daily 5-HT *i.p*. injections at 1 mg/kg and 10 mg/kg doses. For SSRI experiments, sertraline 10mg/kg was injected *i.p.* instead of 5-HT. Tumor cells, 1×10^6^ SKOV3, were injected i.p. into mice, 10 mice per group, 3 days post-initiation of 5HT or sertraline treatments. Daily 5-HT *i.p.* administration continued for the duration of the experiment. Animals were necropsied 4 weeks after tumor cell inoculation. At this time, the entire peritoneal cavity was examined for identifiable disease. Mouse weight, tumor weight, number of nodules and distribution of tumor was recorded by gynecologic oncologists. Tissue was stained for Ki67, a proliferation marker, CD31, an indicator of microvessel density, and caspase 3, an apoptotic marker, and quantified based on number of stained cells per 20x field. Control mice received PBS injections, the solvent used for 5-HT and sertraline.

## SUPPLEMENTARY TABLES


