# Supplementary material for: SSRI use and clinical outcomes in epithelial ovarian cancer
Source: Oncotarget. 2016 Apr 21;7(22):33179–91. doi: 10.18632/oncotarget.8891 (PMC5078084; doi:10.18632/oncotarget.8891)
Supplement: Supplementary file 1 [file oncotarget-07-33179-s001.pdf]

SUPPLEMENTARY TABLES

Supplementary Table S1: Primer sequences (forward and reverse) used for polymerase chain reaction (PCR) to measure mRNA levels in 9 ovarian cancer cell lines

Primer type	Order	Sequence
5HTR1A	Forward	5’-ATCATCATGGGCACCTTCA-3’
5HTR1A	Reverse	5’-GCAGAAGGGCAGAACAAGAG-3’
5HTR1B	Forward	5’-GCTGTCGTCGGACATCACT-3’
5HTR1B	Reverse	5’-AGGGCGATGACACAGAGGT-3’
5HTR1D	Forward	5’-ATTCTGGGGGCCTTTATCAT-3’
5HTR1D	Reverse	5’-AGATGGGGAGGACCAGAGAC-3’
5HTR1E	Forward	5’-CGGGTTCCGAGTGAGACTT-3’
5HTR1E	Reverse	5’-AGGGCCACAGCATTTCTTC-3’
5HTR1F	Forward	5’-AAGTGGGCCCTAGTGAAGGT-3’
5HTR1F	Reverse	5’-AATTTTGGATGGCATTCTGTTT-3’
5HTR2A	Forward	5’-AGTCACTGTACTGCTTTCAGCAA-3’
5HTR2A	Reverse	5’-TGGCACAGAATAATTTAGCACTTT-3’
5HTR2B	Forward	5’-GGAGAAGAAGCTGCAGTATGCTA-3’
5HTR2B	Reverse	5’-GGCAGGACATAGAACAAGTGG-3’
5HTR2C	Forward	5’-CCGAGTCCGTTTCTCGTCTA-3’
5HTR2C	Reverse	5’-TCGCGGGTGTTAGCTGAT-3’

Supplementary Table S2: Univariate cox proportional hazard ratios for antidepressant use, overall survival and PFS in ovarian cancer patients

Covariate	Progression			Death		
	**HR**	**95% CI**	***P***	**HR**	**95% CI**	***P***
Age (unit = 10 yrs)	1.2	1.1-1.4	<0.001	1.3	1.1-1.4	<0.001
Stage *v* 1						
2	1.3	0.6-2.9	0.54	1.2	0.5-2.7	0.63
3	6.2	3.4-11.0	<0.001	4.7	2.6-8.4	<0.001
4	10.2	5.4-19.3	<0.001	7.1	3.8-13.3	<0.001
RD *v* optimal						
Suboptimal	2.9	2.3-3.6	<0.001	2.6	2.1-3.2	<0.001
Grade *v* low						
High	1.3	1.0-1.7	0.04	1.3	1.0-1.6	0.09
Histology *v* other						
Serous	1.6	1.2-2.1	<0.001	1.3	1.0-1.6	0.07
AD *v* non-use						
Use	0.9	0.7-1.1	0.27	0.9	07-1.1	0.37
SSRI *v* non-use						
Use	1.0	0.7-1.3	0.95	0.9	0.7-1.2	0.64

Abbreviations: AD, antidepressant; PFS, progression-free survival; RD, residual disease; SSRI, selective serotonin-reuptake inhibitor; yrs, years.
